# Safety and efficacy of transarterial chemoembolization combined with tyrosine kinase inhibitors and camrelizumab in the treatment of patients with advanced unresectable hepatocellular carcinoma

**DOI:** 10.3389/fimmu.2023.1188308

**Published:** 2023-07-21

**Authors:** Jinpeng Li, Mingxin Kong, Guangji Yu, Song Wang, Zhaozhang Shi, Huihui Han, Yanyan Lin, Jutian Shi, Jinlong Song

**Affiliations:** ^1^ Intervention Ward One, Shandong Cancer Hospital and Institute, Shandong First Medical University and Shandong Academy of Medical Sciences, Jinan, Shandong, China; ^2^ Department of Interventional, Weifang People’s Hospital, Weifang, Shandong, China; ^3^ Department of Interventional, Linyi Cancer Hospital, Linyi, Shandong, China; ^4^ Department of Interventional, Affiliated Hospital of Qingdao University, Qingdao, Shandong, China; ^5^ Department of Oncology, Public Health Clinical Center of Shandong Province, Jinan, Shandong, China; ^6^ Department of Medicine, Jiangsu Hengrui Medicine, Shanghai, China

**Keywords:** camrelizumab, transarterial chemoembolization, tyrosine kinase inhibitors, unresectable hepatocellular carcinoma, therapeutic evaluation

## Abstract

**Objective:**

This study was aimed to evaluate the efficacy and safety of transarterial chemoembolization combined with tyrosine kinase inhibitors and camrelizumab in the treatment of unresectable hepatocellular carcinoma and to explore a new therapeutic strategy for the treatment of advanced HCC.

**Patients and methods:**

A total of 87 patients aged 18-75 years with at least one measurable lesion per Response Evaluation Criteria in Solid Tumors (version 1.1) were included in the study. TACE was administered as needed, and camrelizumab and TKI medication were initiated within two weeks and one week after TACE, respectively. The primary endpoints were progression-free survival and objective response rate.

**Results:**

The 87 patients in this trial were last evaluated on September 28, 2022, and 35.8% were still receiving treatment at the data cutoff. A total of 34 patients (39.1%) died, and the median OS was not reached. The median PFS was 10.5 months (95% CI: 7.8-13.1). The ORR rate was 71.3% (62/87), and the DCR rate was 89.7% (78/87) per mRECIST. According to RECIST version 1.1, the ORR rate was 35.6% (31/87), and the DCR rate was 87.4% (76/87). Ten patients (11.5%) successfully underwent conversion therapy and all achieved R0 resection. Two patients achieved a complete pathological response, four achieved a major pathological response, and four had a partial response. All treatment-related adverse events were tolerated. No serious adverse events were observed, and no treatment-related deaths occurred.

**Conclusions:**

TACE combined with TKI and camrelizumab was safe and effective in treating advanced HCC. Triple therapy may benefit patients with large tumor burden and portal vein cancer thrombus and is expected to provide a new treatment strategy for advanced HCC.

**Clinical Trial Registration:**

ClinicalTrials.gov, identifier ChiCTR2000039508

## 1 Introduction

Hepatocellular carcinoma (HCC) is one of the leading causes of cancer-related deaths worldwide, especially in China, where HCC cases alone account for more than half of the estimated total (1). Most HCC patients have an insidious onset, and about 50% are in the middle and late stages of diagnosis, missing the opportunity for surgical excision. Transarterial chemoembolization (TACE) is a widely accepted treatment for patients with unresectable HCC. However, not all patients are beneficial, and the number of patients with disease progression increases with the number of TACE treatments, with unsatisfactory long-term survival outcomes (2, 3). In recent years, with the application of targeted drugs for liver cancer and the rise of immunotherapy, systematic therapy has made a breakthrough in the treatment of liver cancer. Systemic therapy has been recommended by several guidelines, such as BCLC and Guidelines for the Diagnosis and Treatment of Primary liver Cancer (2022 edition), recommend either a standard treatment for advanced liver cancer or a combination treatment for mid-stage liver cancer (4, 5). Targeted drugs or anti-PD-1 monotherapy have low tumor control rates, high drug resistance rates, and large adverse drug reactions. Therefore, it is urgent to study systematic combination therapy targeting different anticancer mechanisms to reduce the drug resistance rate and improve the efficacy of combination therapy (6). However, ORIENT-32, IMbrave 150, RESCUE, and Keynote-524 studies combined targeted drugs and immunotherapy in the treatment of advanced liver cancer showed a higher ORR rate (20.5%-36%) than single therapy (7, 8). These combination regimens have been recommended by multiple experts as a new first-line treatment option for patients with liver cancer (9).

TACE treatment aggravates hypoxia and immunosuppression of TME, and in addition to local tumor destruction, it has also been shown to have a systemic immune response. For example, PD-1 expression was increased in peripheral mononuclear cells after TACE, and the proportion of CD4+/CD8+ cells was decreased. The main mechanism of ICIs plus TKIs is to improve hypoxia and immunosuppressive tumor microenvironment (TME) by normalizing tumor blood vessels. Therefore, TACE in combination with TKIs and ICIs may theoretically have the potential to further improve the efficacy of uHCC (10, 11). In addition, TACE is the main treatment for advanced liver cancer, but the use of TACE alone has great limitations (12). Local treatment can effectively achieve satisfactory local control; However, it does not always translate into long-term survival benefits. On the other hand, the key to improving long-term prognosis is systemic treatment, but unsatisfactory local control effect will damage the long-term survival advantage of patients. The combination of anti-angiogenic drugs and immune checkpoint inhibitors may overcome the deficiency of TACE and increase efficacy (13). The purpose of this study was to explore the efficacy and safety of three combined treatment modalities.

## 2 Materials and methods

### 2.1 Study design and patients

The prospective study was conducted at five centers in Shandong Province. The study was approved by the Ethics Review Board, and all patients provided written informed consent. All procedures carried out in studies involving human participants complied with the ethical standards of institutional and national research councils, the 1964 Declaration of Helsinki and its subsequent amendments. Eligible patients were aged 18-75 years with unresectable advanced HCC confirmed by histopathology, or diagnostic imaging (CT or MRI) with at least one measurable lesion according to Response Evaluation Criteria in Solid Tumors (RECIST) version 1.1. The performance status of the Eastern Cooperative Tumor Group (ECOG) was 0 or 1, and the Child-Pugh classification was A to B. Patients with the following conditions were excluded: 1) existing immunodeficiency disease or a history of organ transplantation; 2) allergic to camrelizumab active ingredients or excipients; 3) those who participated in other clinical trials; 4) severe cardiopulmonary and coagulation insufficiency, which cannot receive combined therapy with TACE plus targeted drugs and camrelizumab; 5) history of other malignant tumors.

### 2.2 Procedures

During the treatment of TACE, the Seldinger method was used for percutaneous femoral artery catheterization with catheter placed in the abdominal trunk and the common hepatic artery for DSA imaging. The imaging images were collected, including the arterial, parenchymal, and venous phases. In some patients, the superior mesenteric artery, right renal artery, and right phrenic artery were performed to find the blood supply of tumor collateral when necessary. The microcatheter was used to superselect tumor supplying arteries, and all tumor supplying arteries should be superselected successively according to the results of arteriography. The chemotherapy drugs included epirubicin hydrochloride 40-80 mg and oxaliplatin 100 mg. One part of the chemotherapy drugs was injected through the microcatheter, and then the other part was mixed with epirubicin and hydroxycamtocrin and iodide to form an emulsion. At the same time, granular embolization agents (polyvinyl alcohol granules and gelatin sponge granules) were injected alternately for combined embolization. After embolization was complete, angiography was performed again to determine whether the tumor was completely embolized. If there was a local deficiency, embolization was continued until angiography showed complete embolization. Large tumors and multiple tumors in the left and right lobes were treated with TACE twice to prevent liver and kidney failure.

Oral TKI medication should be started within one week after the syndrome was relieved after TACE therapy. The three available TKIs were sorafenib (400 mg, twice a day), regorafenib (80-160 mg, once daily, for 21 days, 7 days off), and lenvatinib. For lenvatinib, patients with a body mass of ≥ 60 kg received an initial dose of 12 mg once a day, and patients with a body mass of < 60 kg received an initial dose of 8 mg once a day. Discontinue TKI medication 3 days before and after TACE treatment. When the patient had a large adverse reaction, the dose of the TKI drug was reduced or taken orally every other day. When adverse drug reactions cannot be tolerated, the medication was discontinued.

Camrelizumab (200 mg intravenously) was administered within two weeks after the syndrome was relieved after TACE treatment and repeated every 3 weeks. If an immunotherapy-related serious adverse event (irSAE) occurred, camrelizumab was discontinued, and immunosuppressants were administered depending on the severity of the complication and the affected organ.

Tumor imaging was conducted at baseline, week 6, week 12, week 18, week 24, and then every 9 weeks thereafter, by contrast-enhanced computed tomography and magnetic resonance imaging. The response was assessed on the basis of the Response Evaluation Criteria in Solid Tumors version 1.1 and Modified solid tumor Evaluation Criteria.

Early combination therapy was defined as treatment with camrelizumab and TKI prior to the first TACE treatment. Late-stage combination therapy was defined as treatment with camrelizumab and TKI after the first TACE treatment. The timing of the use of camrelizumab in combination with TKI was determined by the physician and patient. Excision was performed after a successful descent with sufficient future residual liver (FLR). If researchers observed evidence of clinical benefit, patients may continue the combination therapy after disease progression or be treated with monotherapy.

### 2.3 Outcomes

Efficacy outcomes included complete response (CR), partial response (PR), stable disease (SD), progressive disease (PD), disease control rate (DCR), progression-free survival (PFS), and overall survival (OS). Efficacy was assessed according to Modified solid tumor Evaluation Criteria (mRECIST) and RECIST version 1.1. DCR was defined as the sum of CR, PR, and SD. PFS referred to the time between the initiation of combination therapy and tumor progression or death from any cause. OS referred to the time from the start of combination therapy to the last follow-up or the time of death from any cause.

Adverse events (AEs) were evaluated using the National Cancer Institute Common Terminology Criteria for adverse events version 5.0. Post-embolization syndrome after TACE treatment referred to a series of clinical symptoms such as nausea, vomiting, abdominal pain, fever, and decreased appetite caused by ischemic necrosis of tumor tissue after TACE treatment.

### 2.4 Statistical analysis

All results were statistically analyzed by SPSS 26.0 (SPSS, Chicago, IL, USA). The categorical variable is shown as frequency (percentage) and compared using the Chi-square test or Fisher’s exact test when appropriate. The Kaplan-Meier method was used to estimate the median OS and PFS. A two-sided p-value< 0.05 was considered statistically significant.

## 3 Results

### 3.1 Patient characteristics

A total of 87 patients were enrolled between August 2020 and November 2021 (**Table 1**). All patients received TKI+TACE+ camrelizumab combined therapy. The median age was 56 years (range 34-75), and 81 (93.1%) were male. There were 28 patients (32.2%) with an ECOG performance status 1, 38 (58.6%) with Child-Pugh grade A, 47 (54.0%) with extrahepatic metastases, and 65 (74.7%) with portal vein cancer thrombus. Sixty-nine patients (79.3%) had BCLC stage C, and 75 (86.2%) had hepatitis B infection. Thirty-one (35.6%) patients received sorafenib, 54 (62.1%) patients received lenvatinib, and two patients received regorafenib.

**Table 1:** Baseline characteristics.

Characteristics	TACE plus TKI plus camrelizumab (n=87)
Age	
Median, years (range)	56 (34~75)
<65	70 (80.5%)
≥65	17 (19.5%)
Sex, n (%)	
Male	81 (93.1%)
Female	6 (6.9%)
Median tumor diameter, cm	8.2 (4.8~17.9)
ECOG performance status, n (%)	
0	46 (52.9%)
1	28 (32.2%)
NE	13 (14.9%)
Child-Pugh Class, n (%)	
A	51 (58.6%)
B	29 (33.3%)
NE	7 (8.1%)
CNLC, n (%)	
I	5 (5.7%)
II	14 (16.1%)
III	46 (52.9%)
NE	22 (25.3%)
Portal vein tumor thrombus, n (%)	
Vp0-2	40 (46.0%)
Vp3-4	25 (28.7%)
HBV infection, n (%)	
Yes	75 (86.2%)
No	12 (13.8%)
Extrahepatic metastasis, n (%)	
Yes	43 (49.4%)
No	44 (50.6%)
Number of previous TACE, n (%)	
0	45 (51.7%)
1-2	39 (44.8%)
≥3	3 (3.5%)
AFP (ng/mL), n (%)	
≥400	36 (41.4%)
<400	51 (58.6%)
TKI type, n (%)	
Sorafenib	31 (35.6%)
Lenvatinib	54 (62.1%)
Regorafenib	2 (2.3%)

NE, Not evaluable; CNLC, China liver cancer staging.

### 3.2 Efficacy

**Figure 1:**
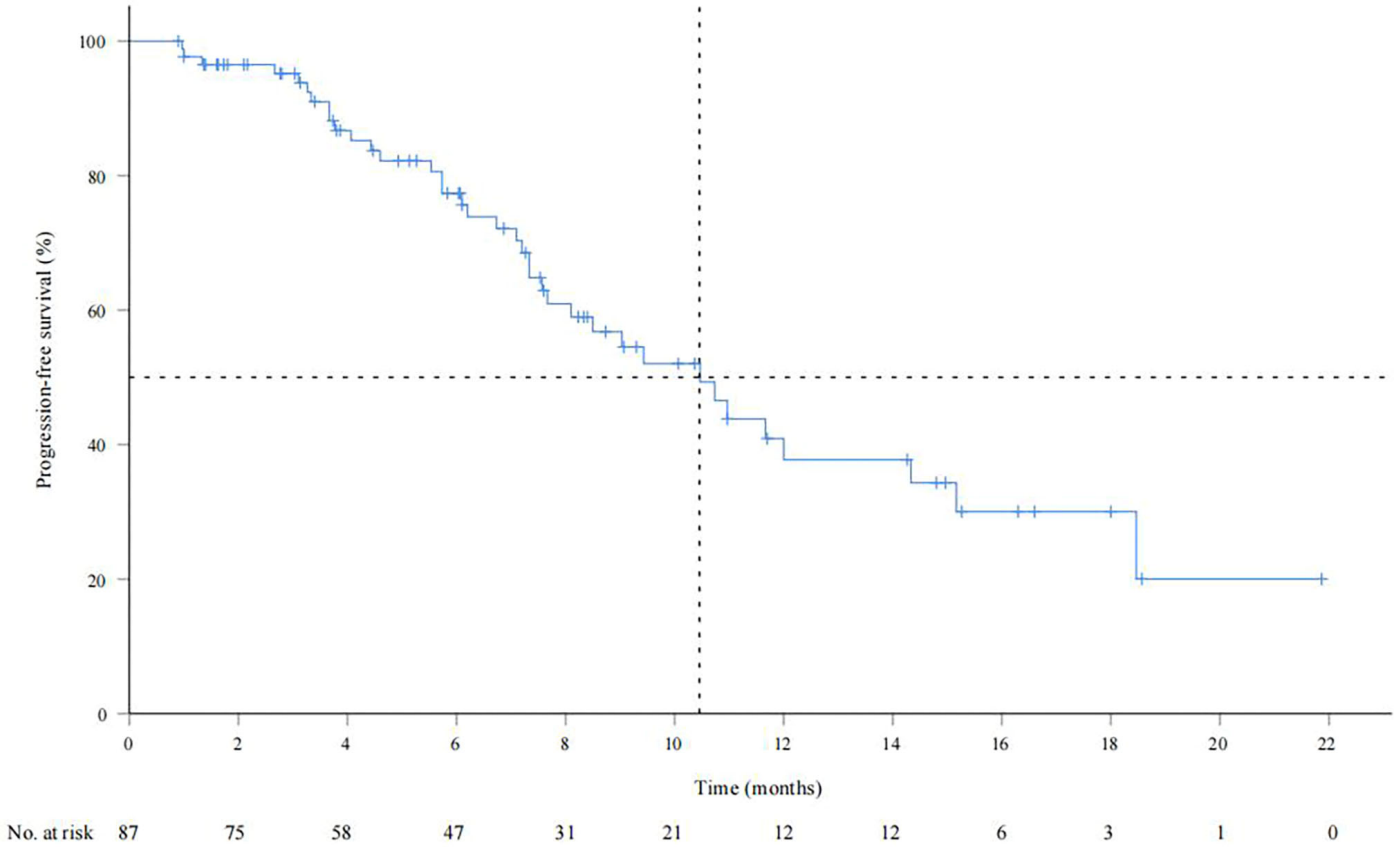
Progression-free survival in the all patients by the Kaplan Meier method per RECIST v1.1.

**Figure 2:**
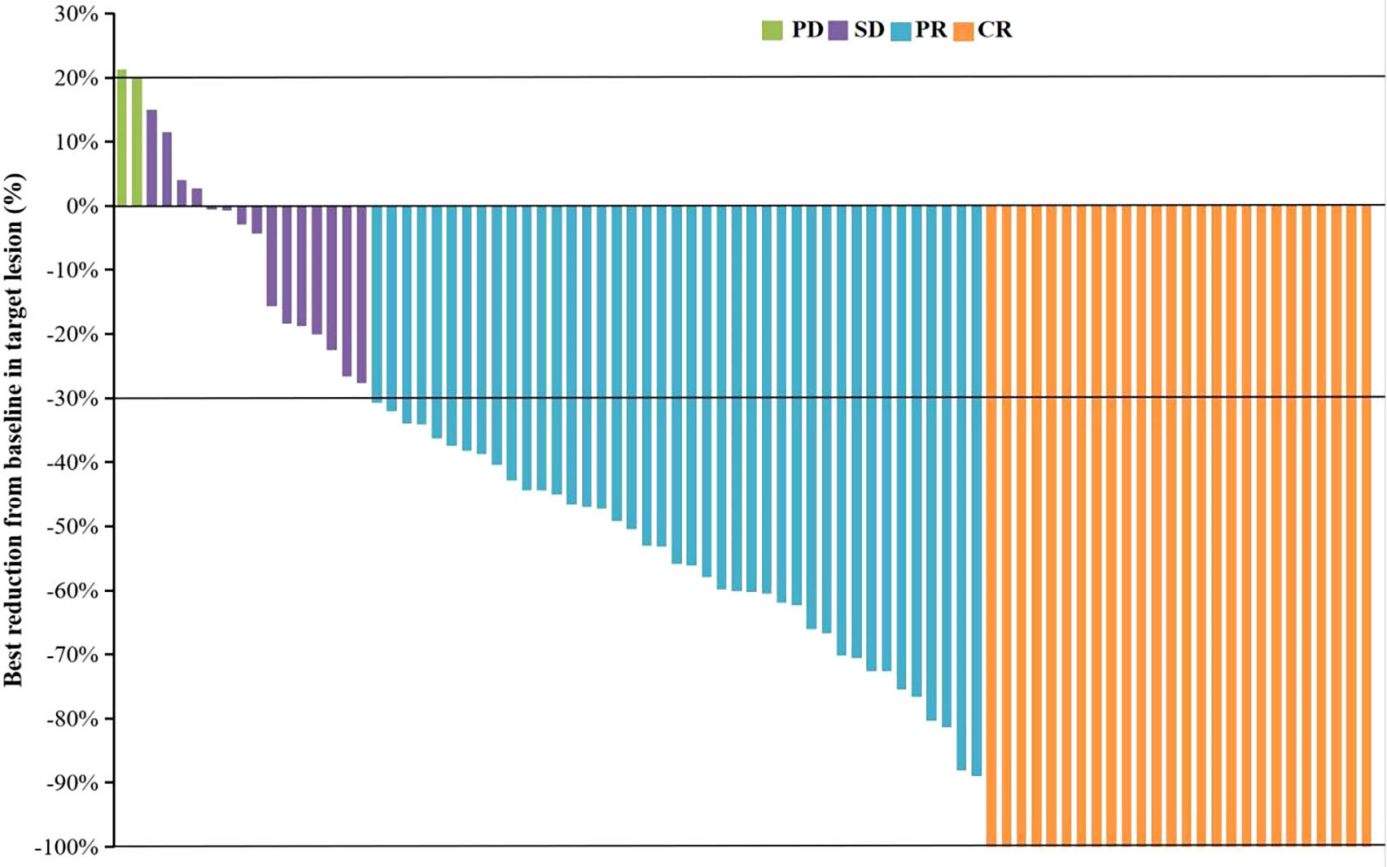
The best change from baseline in the sum of the target lesion diameter per patient. CR, complete response; PR, partial response; SD, stable disease; PD, progressive disease.

**Figure 3:**
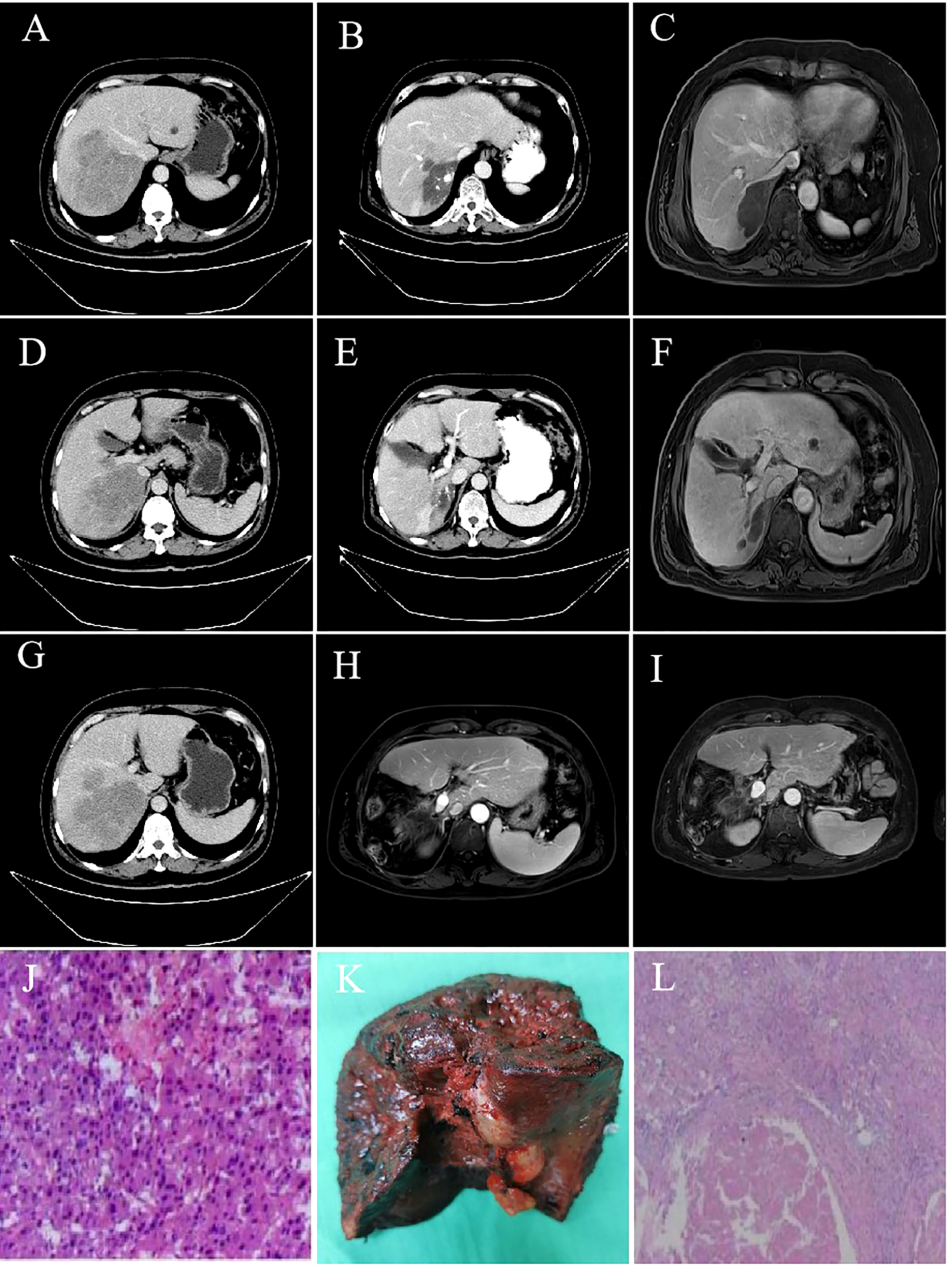
Imaging data and postoperative pathology of a 55-year-old male patient with advanced liver cancer after successful conversion therapy. **(A, D, G)** hcc with main carcinoma thrombus of the right portal vein; **(B, C, E, F)**: After 2 TACE combined with camrelizumab and TKI treatment for five cycles, liver lesions shrank, and portal vein cancer thrombus retreated; **(H)**: No intrahepatic recurrence or metastasis one month after resection; **(I)**: No intrahepatic recurrence or metastasis in 20 months after resection; **(J)**: Pathological results of baseline biopsy; **(K)**: Postoperative pathological specimens; **(L)**: The pathological results of the lesion resection after combination therapy showed that the tumor and cancer thrombus were completely necrotic, no cancer cells were detected, inflammatory cell infiltration and interstitial fibrosis reached pathologic complete response.

As of September 28, 2022, the median duration of follow-up was 13.6 (0.83-24.9) months. A total of 34 patients (39.1%) died, and the median OS was not reached. The OS rates of 6, 12 and 18 months were 84.6%, 73.4% and 55.2%, respectively (**Supplementary Figure 1**). The median PFS was 10.5 months (95% CI: 7.9-13.1) (**Figure 1**). According to mRECIST, the ORR rate was 71.3% (62/87) and the DCR rate was 89.7% (78/87), including 16 patients with CR, 46 patients with PR, 16 patients with SD, and six patients with PD. According to RECIST1.1, the ORR rate was 35.6% (31/87) and the DCR rate was 87.4% (76/87); there were 31 patients with PR, 45 with SD, and eight with PD, and none with CR (**Table 2**). Compared to the baseline, the target lesion burden decreased in 78 (89.7%) patients (**Figure 2**). Ten of the patients (11.5%) successfully underwent conversion therapy. All patients achieved R0 resection. There were no Clavien-Dindo III or higher complications. Common postoperative complications such as pain and elevated aminotransferase were effectively controlled after symptomatic management. Postoperative pathology reports included two complete pathological responses (cPR), four pathological responses (MPR), and four partial responses (pPR) (**Figure 3**).

**Table 2:** Response to combined therapy.

Tumor response	RECIST1.1 n (%)	mRECIST n (%)
CR	0	16 (18.4%)
PR	31 (35.6%)	46 (52.9%)
SD	45 (51.7%)	16 (18.4%)
PD	8 (6.1%)	6 (6.9%)
NE	3 (3.4%)	3 (3.4%)
ORR(CR+PR)	31 (35.6%)	62 (71.3%)
DCR(CR+PR+SD)	76 (87.4%)	78 (89.7%)

CR, Complete response; PR, Partial response; SD, Stable disease; PD, Progressive disease; NE, Not evaluable; ORR, Objective response rate; DCR, Disease control rate.

**Figure 4:**
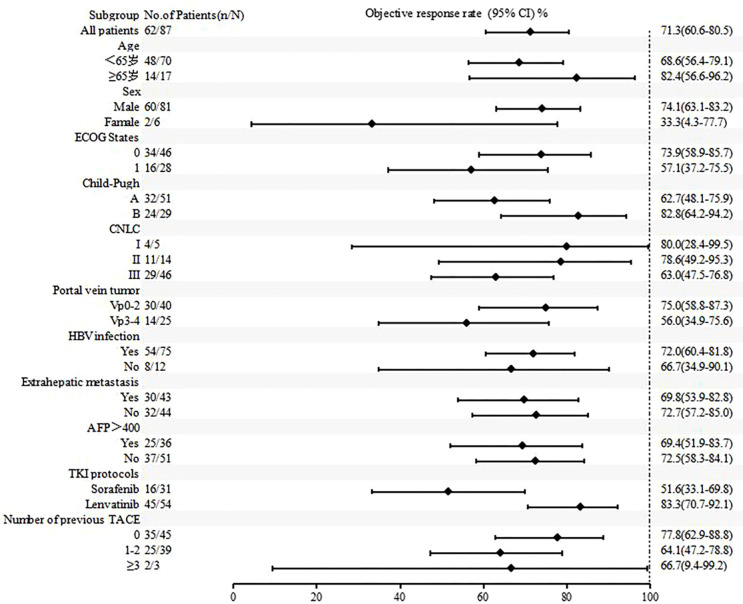
Subgroup analysis of objective response rate according to baseline characteristics.

**Figure 5:**
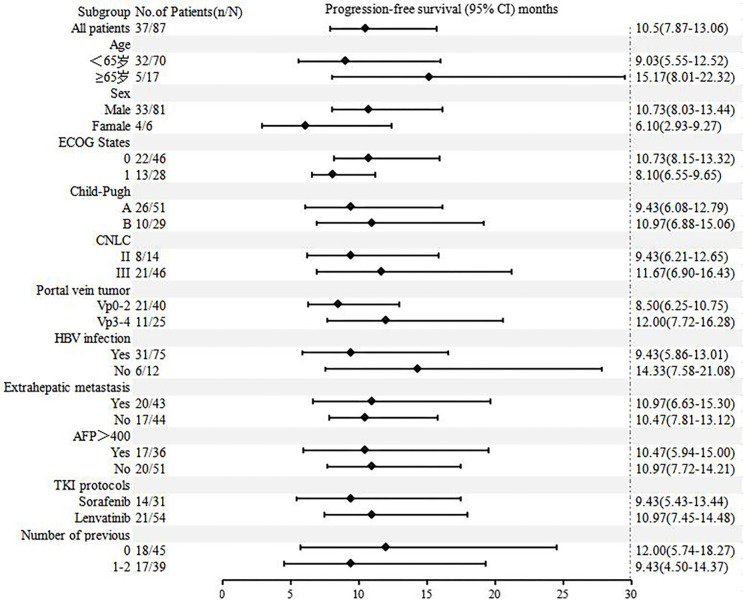
Subgroup analysis of progression-free survival according to baseline characteristics.

**Figures 4** , **5** show the analysis of ORR and PFS in different subgroups. Poisson regression with robust error variance calculated treatment effectiveness and 95% confidence intervals. The ORR and PFS showed consistent benefits in subgroups based on ECOG score, HBV infection, baseline alpha-fetoprotein level, combined TKI, and the number of TACE treatments. In addition, the combination of triple therapy with lenvatinib was more beneficial than that with sorafenib, and there was a trend toward benefit among the TACE-naïve group as compared with the previous TACE-treated group. Late-stage combination therapy significantly improved PFS than early combination therapy (**Supplementary Table 1**).

### 3.3 Safety

After enrollment, the median number of interventional therapy was two times (1-9 times), and the cumulative total number of interventional therapy was 203 times. The median treatment period for camrelizumab was 6 cycles (range 1-25). The adverse reactions during the observation period are shown in **Table 3**. A total of 87 patients (100%) who received combination therapy developed at least one treatment-related AE. The most common AEs were hypoproteinemia (80 cases), elevated lactate dehydrogenase (70 cases), elevated glutamic oxalacetic transaminase (69 cases), elevated bilirubin (68 cases), abdominal pain (54 cases), nausea (29 cases), and RCCEP (23 cases). The incidence of grade 3-4 adverse reactions was 67.8%, and no treatment-related deaths occurred. After three cycles of camrelizumab combined with lenvatinib, one patient developed liver failure, which was cured and stopped targeted therapy and immunotherapy after prednisolone and hepatoprotective therapy. Related AE symptoms or signs were relieved or eliminated after symptomatic treatment, dose reduction, or interruption of medication.

**Table 3:** Adverse events.

Adverse events	Any Grade		Grades 3-4	
	N	%	N	%
Hematological toxicities				
Hypoalbuminemia	80	92.0	1	1.1
Binding bilirubin increased	68	78.2	11	12.6
Lymphocyte count decreased	66	75.9	31	35.6
Blood bilirubin increased	64	73.6	9	10.3
Lactate dehydrogenase increased	70	80.5	0	0
Platelet count decreased	65	74.7	15	17.2
Alanine aminotransferase increased	53	60.9	10	11.5
Aspartate aminotransferase increased	69	79.3	14	16.1
Anemia	52	59.8	9	10.3
White blood cell count decreased	43	49.4	7	8.0
Neutrophil count decreased	45	51.7	9	10.3
Nonhematological toxicities				
Reactive cutaneous capillary endothelial proliferation	23	26.4	5	5.7
Nausea	29	33.3	0	0
Pyrexia	21	24.1	0	0
Abdominal pain	54	62.1	8	9.2
Vomiting	13	14.9	0	0

## 4 Discussion

TACE is currently the standard first-line treatment for mid-stage HCC recommended by various guidelines around the world (4, 14). It is also a common modality of palliative treatment for advanced HCC. However, mid-stage and late-stage HCC has a large tumor burden and is characterized by intratumoral heterogeneity, often accompanied by portal vein cancer thrombus and arteriovenous fistula, which often require multiple TACE treatments in a short period of time. After TACE treatment, the tumor often makes it difficult to achieve pathological complete necrosis (15). In addition, for large liver cancer or massive liver cancer, repeated TACE will cause serious damage to liver function. Still, after these treatments fail, there is a lack of corresponding remedial measures (16). Based on this situation, it is necessary to explore effective treatment programs based on TACE (17).

In recent years, immunotherapy has achieved the obvious curative effect in the field of liver cancer (18), while TACE combined immunotherapy has strong rationality in theory, which can directly kill or inhibit the growth of tumor cells, destroy the release of tumor antigen substances by tumor cells, enhance the immune effect, and thus improve the curative effect and prolong the survival of patients. In addition, targeted combination immunotherapy can synergically enhance the efficacy of TACE, and TKI combined immunotherapy will help eliminate the factors of tumor recurrence caused by tumor angiogenesis after TACE (19). Although PD-1 immunotherapy has shown promising efficacy in the treatment of HCC, its efficacy is still less than 20% when used alone. IMbrave 150 and RESCUE studies confirmed that target-free therapy showed good efficacy and safety in first-line or second-line therapy for advanced HCC, providing a new treatment option for unresectable HCC (6, 7, 20). In these clinical studies, most patients received local therapy, including TACE (21), but there were few studies on TACE combined with anti-angiogenic therapy and anti-PD-1 antibody.

This study evaluated the efficacy and safety of TACE + TKI + PD-1 antibody in the treatment of unresectable HCC and explored the factors influencing its prognosis. The median OS was not reached, and the median PFS was 10.5 months (95% CI 7.8-13.1), the ORR was 35.6% and the DCR was 87.4% (RECIST version 1.1), higher than the previous PD-1 inhibitors or TKI monotherapy, and higher than RESCUE study results (19). Apatinib combined with camrelizumab for first-line and second-line treatment of unresectable HCC had PFS of 5.7 and 5.5 months, respectively, and the ORR of first-line treatment in the phase 2 RESCUE study was 34.3%. In the IMbrave150 trial, 48% of patients in the atezolizumab plus bevacizumab group received prior topical therapy, while more than 60% of patients in the RESCUE trial received interventional therapy. This study showed that TACE combined with TKI and camrelizumab in the treatment of unresectable HCC patients had higher overall survival and tumor response rates, suggesting that TACE combined with targeted therapy and immunotherapy is a promising treatment option. The possible reason is that TACE can cause ischemic tumor necrosis, thus reducing tumor burden, resulting in tumor tissue release of tumor antigen, increased expression of PD-1 and PD-L1, and improved tumor recognition. Anti-vascular endothelial growth factor (VEGF) therapy normalizes the tumor vasculature, reduces additional VEGF-mediated immunosuppression in the tumor and its microenvironment, and promotes T-cell infiltration (22, 23). Therefore, the combination of TACE, TKI, and camrelizumab may produce synergistic antitumor effects and improve clinical outcomes in patients with unresectable liver cancer.

Subgroup analyses of ORR and PFS based on baseline characteristics found that combination with lenvatinib showed better benefits than combination with sorafenib in the triple therapy, which was consistent with the results of the REFLECT study (24). Lenvatinib showed a trend of benefits compared with sorafenib in both alone and combined therapy. In addition, those who did not receive TACE treatment in the past also tended to benefit more than those who did. This may be due to repeated TACE treatments damaging liver function and embolic hypoxia increasing VEGF, which can promote tumor recurrence and metastasis (22). The PFS of late combined target therapy with TACE was longer than early combined therapy, which may be related to the time-consuming process of tumor ischemia-hypoxic necrosis releasing large amounts of antigen and altering the tumor microenvironment after TACE.

Tumor burden and portal vein cancer embolism are factors for poor prognosis of patients with advanced liver cancer. Effective therapeutic approaches must be explored to control tumor progression, reduce tumor volume, and improve patient prognosis. Therefore, transformation therapy has become a research hotspot for unresectable HCC and middle and advanced HCC (25). Although neoadjuvant therapy for colorectal cancer, lung cancer, breast cancer, and other tumors is relatively common, due to the insensitivity of HCC to traditional chemotherapy and radiotherapy, neoadjuvant therapy and transformation therapy have not made breakthrough progress in HCC. In this study, 74.7% of patients had portal vein cancer thrombus, and 28.7% had portal vein main cancer thrombus. In the treatment process of TACE combined with TKI and PD-1 antibody, ten (11.5%) patients underwent surgery. Notably, two patients achieved pCR, four patients achieved MPR. Eight patients with obvious lipiodide deposition and cancer thrombus retracting to secondary branches, which achieved the purpose of downstaging. None of the patients had serious complications such as liver failure, gastrointestinal bleeding, pulmonary embolism, acute renal failure, severe infection, and bile duct infarction. The results of this study demonstrated the safety and efficacy of TACE combined with targeted therapy and immunotherapy for HCC with portal vein thrombus.

The safety profile of triple therapy was consistent with those of the individual drugs. No new safety signals were observed. But all of these adverse events are generally manageable. Notably, compared with camrelizumab monotherapy, the incidence of RCCEP was reduced in combination therapy, which may be due to the involvement of VEGF signaling pathways in the mechanism of RCCEP, and other studies reported similar findings (26).

There are several limitations to this study. First, the sample size of this study is relatively small, which may reduce the statistical efficacy. Second, the follow-up time in this study was short, and a longer follow-up time was needed to verify the further OS. Third, there were few cases in the combination timing group, and finding the right combination therapy timing is still necessary.

## 5 Conclusion

In summary, TACE combined with TKI and camrelizumab has longer progression-free survival benefits and a high tumor control rate with a manageable safety profile in the treatment of advanced liver cancer. Thus, the combination regimen could provide a new treatment option for this patient population.

## Data availability statement

The raw data supporting the conclusions of this article will be made available by the authors, without undue reservation.

## Ethics statement

The studies involving human participants were reviewed and approved by China Ethics Committee of Registered Clinical Trial. The patients/participants provided their written informed consent to participate in this study.

Written informed consent was obtained from the individual(s) for the publication of any potentially identifiable images or data included in this article.

## Author contributions

JLS and JL conceived and designed the study. JLS, JL, MK, GY, SW, and ZS enrolled patients and collected the data. HH and YL analyzed the data. All authors participated in data interpretation. All authors contributed to the article and approved the submitted version.
