# Supplementary material for: Safety and efficacy of transarterial chemoembolization combined with tyrosine kinase inhibitors and camrelizumab in the treatment of patients with advanced unresectable hepatocellular carcinoma
Source: Front Immunol. 2023 Jul 21;14:1188308. doi: 10.3389/fimmu.2023.1188308 (PMC10401037; doi:10.3389/fimmu.2023.1188308)
Supplement: Supplementary file 1 [file DataSheet_1.pdf]

Supplementary Material

Safety and efficacy of transarterial chemoembolization combined with tyrosine kinase inhibitors and camrelizumab in the treatment of patients with advanced unresectable hepatocellular carcinoma

Jinpeng Li[1], Mingxin Kong[2], Guangji Yu[3], Song Wang[4], Zhaozhang Shi[5], Huihui Han[6], Yanyan Lin[6], Jutian Shi[1], Jinlong Song[1]

*** Correspondence:** Jinlong Song: jls7286@163.com

Supplementary table 1. Influence of joint timing on PFS

	mPFS（95% CI)	HR（95% CI）	P-value
Early combination	7.2 (3.0-11.4)	0.285 (0.124-0.655)	0.003
Late combination	11.7 (9.9-13.5)		

Early combination: treatment with camrelizumab and TKI prior to the first TACE treatment. Late combination: treatment with camrelizumab and TKI after the first TACE treatment. PFS: progression-free survival

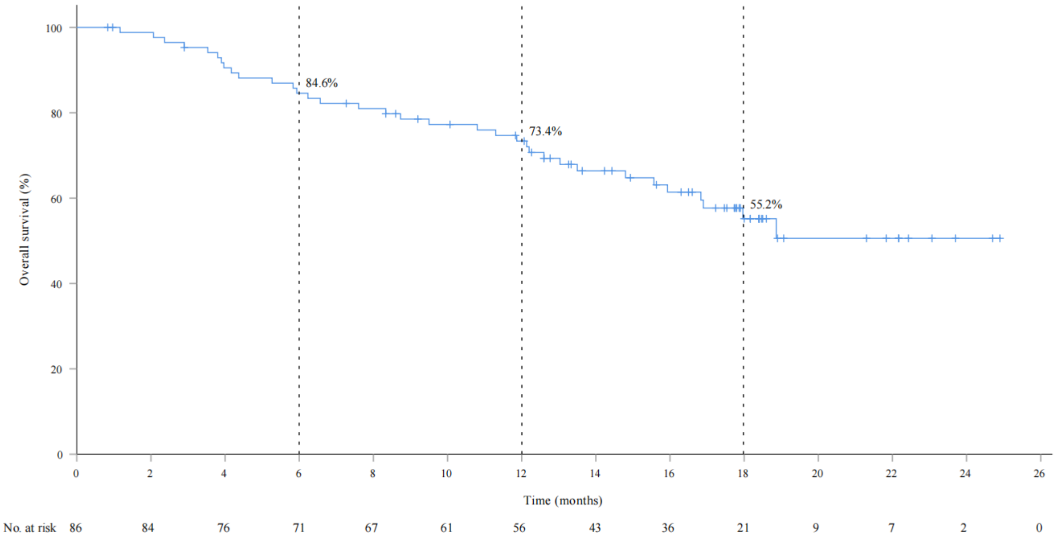

Supplementary figure 1. Overall survival in the all patients by the Kaplane-Meier method per RECIST v1.1
